# Is it Possible to Mechanical Recycle the Materials of the Disposable Filtering Masks?

**DOI:** 10.3390/polym12112726

**Published:** 2020-11-17

**Authors:** Daniele Battegazzore, Fulvia Cravero, Alberto Frache

**Affiliations:** Dipartimento di Scienza Applicata e Tecnologia, Politecnico di Torino, Alessandria site, Viale Teresa Michel 5, 15121 Alessandria, Italy; fulvia.cravero@polito.it (F.C.); alberto.frache@polito.it (A.F.)

**Keywords:** filtering face mask, recycling, personal protective equipment (PPE), covid-19, disposable, non-woven fabric, recycled-polypropylene (rPP)

## Abstract

In a singular period, such as during a pandemic, the use of personal protective masks can become mandatory for all citizens in many places worldwide. The most used device is the disposable mask that, inevitably, generates a substantial waste flow to send to incineration or landfill. The article examines the most diffused type of disposable face mask and identifies the characteristic of the constituent materials through morphological, chemical, physical, and thermal analyses. Based on these investigations, a mechanical recycling protocol with different approaches is proposed. Advantages and disadvantages of the different recycling solutions are discussed with considerations on necessary separation processes and other treatments. The four solutions investigated lead to a recycling index from 78 to 91% of the starting disposable mask weight. The rheological, mechanical, and thermo-mechanical properties of the final materials obtained from the different recycling approaches are compared with each other and with solutions present on the market resulting in materials potentially industrially exploitable.

## 1. Introduction

In a study published by Politecnico di Torino, it was estimated that 1 billion face masks are needed for the Italian population each month if a complete reopening of the companies is maintained [1]. One billion masks weighing at least 3 g turn into 3000 tons of waste every month, equal to 2.6% of the national plastic collection (Urban Waste Report 2019—Ispra [2]).

Not all states behaved similarly during the pandemic, according to the data collected from Ipsos and published on Statista website [3,4], but it was found that over 50% of the population interviewed in another survey by Imperial College London and YouGov wear the commonly called “medical face mask”, thus, a disposable device [5].

**Figure 1:**
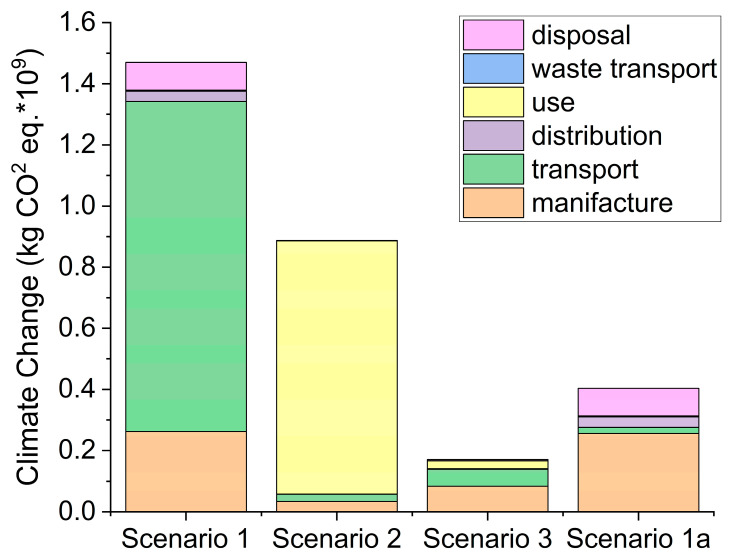
Climate change (kg CO_2_ eq.) impact results for each face mask scenarios. Scenario 1—Single-Use Masks; Scenario 2—Reusable Masks (Manual Washing); Scenario 4—Reusable Masks (Machine Washing); Scenario 1a—Single-Use Masks manufactured in Turkey; all data from Reference [6].

In this scenario, an interesting study on the environmental impact of different face mask-adoption in UK has been presented by Allison et al. [6]. This study assumed the use of one disposable mask per person per day (scenario 1) or reusable masks used in rotation for a year. In a first rotation option (scenario 2), an individual has only two masks for a year, and it was assumed that manual washing is used due to the necessity of every day washes. In the last scenario, scenario 3, four masks are used for a year and washed with laundry every two days. Life cycle impact assessment (LCIA) results showed that the scenario in which four masks are used in rotation and machine-washed (scenario 3) has the lowest environmental impact in all of the impact categories analyzed except water scarcity, where single use mask is the best solution (1.40 × 10^8^ m³ world equiv.) [6]. For the climate change index, the disposable mask (one each day for a year) is the worst solution (Scenario 1: 1.47 × 10^9^ kg CO_2_ eq.) (Figure 1). Nevertheless, around 74% of kg equivalent of CO_2_ are due to the transportation of the masks from China (green part in Figure 1). In the event that the manufacturer and materials come from the same country or neighboring ones as the users, it would be the second best choice (Figure **1** Scenario 1a: 0.4 × 10^9^ kg CO_2_ eq.), not so far from the best solution (Scenario 3: 0.17 × 10^9^ kg CO_2_ eq.) [6]. Furthermore, the use of only 4 recyclable masks in a year seems a bit underestimated, and just doubling or tripling this number would bring the two scenarios (3 and 1a) really close to each other.

It is undeniable from this report that the impact of the single use mask in the disposal (pink part in Figure 1 histograms) remains one of the biggest problems of this solution (22% of the kg CO_2_ eq. in Scenario 1a [6]).

For this reason, to limit the impact on the environment due to the disposal of the single use masks, they have to be recycled. Unfortunately, this situation has not been previously hypothesized due to the lack of need: indeed, before the pandemic, the masks were used only in a restricted work area or in the medical environment in a small amount [7]. Actually, it is absolutely necessary to find a different solution to that of landfilling or incineration [8,9]. In addition, in order to be able to recycle the used masks, the origin and safety in their handling is fundamental, as well as the local legislations ordinance.

Theoretically, the disposable mask could be disinfected and sterilized for direct reuse [10,11,12,13], but possible other contaminations and reduction in the filtering effectiveness make it difficult and not recommended by scientific technical committees. A second solution is to recycle the materials of which the masks are made, which is the central focus of this article.

When the masks end their use as personal protective equipment (PPE), according to the legislation of many countries, including Italy, they become a “municipal waste” and are conferred together with the other unsorted household waste (Istituto Superiore di Sanità (ISS) COVID-19 Report n. 26/2020 [14]). In the same text, it is also considered reasonable to assign the non-dangerous code in consideration of the fact that these masks are generally used for prevention by healthy people. Furthermore, regarding the survival of the virus, the same document reports that the viruses can survive from 48 h up to 9 days, depending on the material, concentration, temperature, and humidity.

It is therefore apparent from a medical point of view that, even if the masks were from infected subjects, the virus would no longer persist after 10 days. Any further treatment for disinfection would have to be deeply evaluated but already studied by several researchers [10,12,13,15,16].

In the Italian regulation, there is no plan for the recycling of these materials, except their transfer to landfills/incinerators as undifferentiated material. In addition, the scientific literature has never investigated a possible recycling strategy of the non-woven fabrics constituting the masks. For these reasons, the research presented is certainly innovative, necessary, and urgent.

The paper provides the first attempt to define and validate a material recycling protocol and subsequent possible material exploitation in uses other than that of origin. In this article, the validation of the recycling idea was carried out using only new disposable face masks, but it should be the basis for the use of disinfected masks after use by a separate collection circuit that some municipalities want to implement. Obviously, many producers and many types of masks are present on the market, and an exhaustive investigation of everything that may be present is extremely difficult (e.g., 94 companies and 196 products present in an online database [17] and other researches [13,18,19]).

The separation and grinding of the mask components (face filter, ear loop, metal material in the nose part if present) was performed manually but can be industrialized as in the separate collection of plastic with flotation and/or metal separation as done for solid plastic wastes [20].

The parts that float are definitely made of polymers, thus being potentially fully recyclable. The parts that sink must be adequately separated for different densities as they can contain different polymers, natural fabrics, and also metals. Each component can be used in its own recycling chain, if separated, or a mixture of the various materials can also be hypothesized [20]. Obviously, an economic evaluation would have to be carried out for the most sustainable solution.

In the first part of this study, the mask materials were identified and morphologically, chemically, and thermally characterized with Scanning Electron Microscopy (SEM), Energy Dispersive X-ray Spectrometry (EDX), Attenuated Total Reflection (ATR), Differential Scanning Calorimetry (DSC), and Thermogravimetric Analysis (TGA).

A central section of the article is dedicated to the description of the recycling choices made. What turned out to be thermo-plastic material from this screening was directly used in a laboratory extruder for plastics in different ways for the production of pellets.

Subsequently, thin films samples by compression molding were produced from the extruded pellets. The morphological, mechanical, and thermo-mechanical properties of the different recycled materials were evaluated and compared with commercially available ones.

## 2. Materials and Methods

### 2.1. Materials

The disposable face masks were purchased from Jointown International from Jinan Shangrun Tongda Composite Material Co., Ltd. (Jinan, China), Standard GB/T 32610-2016, and are coded as MCA in the paper.

The total weight of one face mask equipment is 3.24 g (average of 10 masks): 78.6% for the filtering face mask (FM), 12.6% for the ear loop (EL), and 8.8% for the nose wire (NW).

Two other types of masks were compared: coded as MCD—produced by Oxymore Hangzhou Yuhong Sanitary Products Co. (Hangzhou, China), Ltd.; and coded as MCP—produced by Hubei Hongkang Protective Products Co., Ltd. (Xiantao, China).

### 2.2. Mask Recycling Process

The three main components of the masks (face mask (FM), ear loop (EL), and nose wire (NW)) were manually separated from each other and grinded with the aid of scissors. The FM protective layers were cut in squares of 10–20 mm in size and the EL cut in length less than 10 mm for the feeding in the extrusion machine. The NWs were not recycled in this study.

The melt mixing was assessed in a co-rotating twin screw micro extruder DSM Xplore 15 mL microcompounder (Geleen, The Netherlands). The screw speed was maintained at 50 rpm for the feeding time and increased to 100 rpm for the residence time fixed for all runs at 2 min. The heating temperature was selected at 190 °C or 230 °C. The extruded material was manually pelletized and placed inside a mold made of a 0.1 mm thickness aluminium foil with a 100 × 100 mm^2^ hole inside two metal plates. Using a hot compression molding press (Collin P 200 T press, Maitenbeth, Germany) at 180 or 230 °C for 2 min under a pressure of 5 MPa, the thin film were obtained. The same compression molding conditions were adopted to obtain disks with a diameter of 25 mm and a thickness of 1 mm for rheology tests.

### 2.3. Methods for Analyses

The morphology of the different parts of the masks and the cross sections of the extruded materials were observed after gold-metallization using a EVO 15 Scanning Electron Microscope (SEM) from Zeiss (beam voltage: 20 kV working distance: 8.5 mm, Oberkochen, Germany). The fracture surfaces were obtained from cryogenic fracture in liquid nitrogen. Elemental analysis was carried out by EDX (Energy Dispersive X-ray spectroscopy) using an X-ray probe (Oxford Ultim Max, model 40, High Wycombe, UK).

Attenuated Total Reflection (ATR) was used to identify the materials of the different parts of the masks using a Frontier FT-IR spectrophotometer (16 scans and 4 cm^−1^ resolution, Perkin Elmer, Waltham, MA, USA) equipped with a universal ATR sampling accessory and a diamond crystal.

The thermal properties of mask parts were evaluated by Differential Scanning Calorimetry (DSC) and Thermogravimetric Analysis (TGA).

DSC measurements were performed by a DSC TA Q20 (TA Instruments, New Castle, DE, USA), using 8 ± 1 mg of sample, and the chamber was purged by nitrogen. Each sample was heated from −50 to 250 °C at 10 °C/min. The melting temperature, as well as the melting enthalpy, was obtained from the peak maximum and as the integral of the area under the heat flow curve, respectively. In order to evaluate the crystallinity percentage of the polypropylene (PP)-based pellets, the value of 208 J g^−1^ [21] was considered as reference for the 100% crystalline PP melting enthalpy.

Thermogravimetric Analyses (TGA) were carried out in air, from 50 to 90 °C with a heating rate of 10 °C/min followed by a 60-min isotherm. Subsequently, a second ramp (10 °C/min) brought the temperature to 190 °C or 230 °C, and a second isotherm for 30 min was assessed. To conclude the test, a third ramp (10 °C/min) from the isothermal temperature up to 700 °C was done. The used equipment was a Discovery thermo balance (TA Instruments, New Castle, DE, USA) (experimental error: ± 0.5 wt.%, ± 1 °C) with samples of approximately 10 mg placed in open alumina pans and fluxed with air at 25 mL/min.

The rheological properties of the melt blended materials were measured using an ARES rheometer (TA Instruments, New Castle, DE, USA) fitted with a 25-mm parallel plate geometry. The gap between the plates was set to 1 mm. Dynamic strain sweep tests were carried out to confirm the linearity of the viscoelastic region up to 10% strain at 100 rad/s frequency. Frequency sweeps were carried out to determine the complex viscosity (η*) over a frequency range of 0.1–100 rad/s at 10% strain. Tests were performed under a nitrogen atmosphere to avoid any degradation.

Tensile tests were performed at room temperature using a loading cell of 50 N (error <0.25%), a strain rate of 1 mm/min, and a gauge length of 20 mm with an Instrom 5966 model machine (Norwood, MA, USA) equipped with 250 N rubber face pneumatic grips. The specimens for the stress–strain analyses were 40 × 10 × 0.1 mm^3^ obtained by cutting the compression-molded films with scissors. Three specimens were used for each formulation, and the average values and corresponding standard deviations of the tensile modulus (E), elongation at break (ε) and maximum tensile strength (σ_M_) were calculated and reported.

Dynamic-mechanical thermal experiments (DMTA) were performed using a DMA TA Q800 (TA Instruments, New Castle, DE, USA) with tensile film clamp. The analyses were performed on 30 × 6 × 0.1 mm^3^ samples cut with a scissors from the compression molded film. The temperature range was set from 30 °C to 120 °C, the heating rate at 3 °C/min, and the frequency at 1 Hz. The tests were performed in strain-controlled mode with 0.05% of deformation amplitude and 0.05 N of preload force. All tests were made according to the ISO 6721 standard. Two specimens were used for each formulation.

The samples for the mechanical tests were conditioned at 23 °C and 50% of relative humidity (R.H.) before analyses.

## 3. Results and Discussion

### 3.1. Disposable Mask Characterization

#### 3.1.1. Morphological Analyses

**Figure 2:**
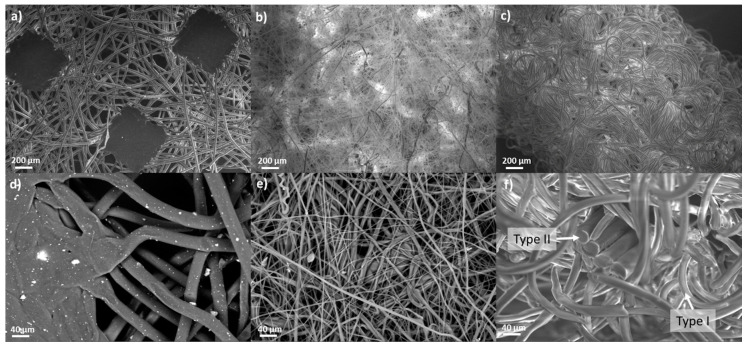
SEM magnification of: (**a**,**d**) inner part of the face mask (FM); (**b**,**e**) central filtering layer of FM; (**c**) ear loop (EL) and (**f**) EL section.

SEM images of the different parts of the MCA mask can be seen in Figure 2. The inner layer has a non-woven fabric morphology (Figure 2a,d) and is made of fibers with uniform circular section of approximately 40 µm. These fibers are stuck together, thanks to square melting junctions at regular distances of few hundreds of µm (Figure 2a). Moreover, in the backscattered electron image in Figure 2d, the presence of a filler with the dimension of few micrometers can be appreciated as white dots. The EDX analysis on that particles reveals the presence of 22.0 ± 0.7 wt.% of Ca, 29.0 ± 0.8 wt.% of O, and 47.7 ± 0.7 wt.% of C elements. It is likely to identify this filler as calcium carbonate considering that the amount of C element could be affected by the presence of the polymer matrix and that the ratio between Ca and O is only slightly lower than the stoichiometric one in CaCO_3_. The outer layer of the mask is exactly the same of the inner one, in terms of fibers morphology, melting junctions, and presence of calcium carbonate as filler.

On the other hand, the filter layer of FM (Figure 2b) can be distinguished from the former ones (Figure 2a) for the different structure in terms of shape and dimension of the fibers. As can be seen in Figure 2e, the diameter varies from a few µm to some tens of µm, and it changes along to the main axis of each one. Contrary to what described for the inner and outer layer, no filler was found in the filter part.

The morphology of the EL was then examined. It can be seen the surface of the sample in Figure 2c and the section at increased magnitude in Figure 2f. Two families of fibers can be distinguished: the main structure is made of thinner fibers (named Type I) waved with a loose pattern in which a second system of fiber (named Type II) is embedded. The section of the Type I is, on average, 19 µm, while, for Type II, the dimension of each fiber is about 37 µm. In addition, as can be seen in Figure 2f, the Type II is organized in bundles of single parallel fibers running straight along the ear loop. The EDX analysis reveals a different concentration of nitrogen element in the two families of fibers: for Type I, it is 11.8 ± 2.6 wt.%, while, for Type II, the value is 5.5 ± 1.3 wt.%. Lastly, there are also embedded in the section particles with an average diameter lower than 1 µm, containing titanium and aluminium (supporting information Figure S1).

In addition, the NW has been analyzed (supporting information Figure S2): It is made of a metal wire covered by a polymer layer. The nature of the metal has been characterized with EDX analysis that reveal the presence of an iron core covered with a zinc layer (> 60 wt.%). The galvanization is a common process intended to prevent the corrosion of the iron. The action of this protective layer is further improved by the presence of the polymeric cover.

#### 3.1.2. Chemical Analyses

The different parts of the disposable mask have been separately characterized with ATR.

**Figure 3:**
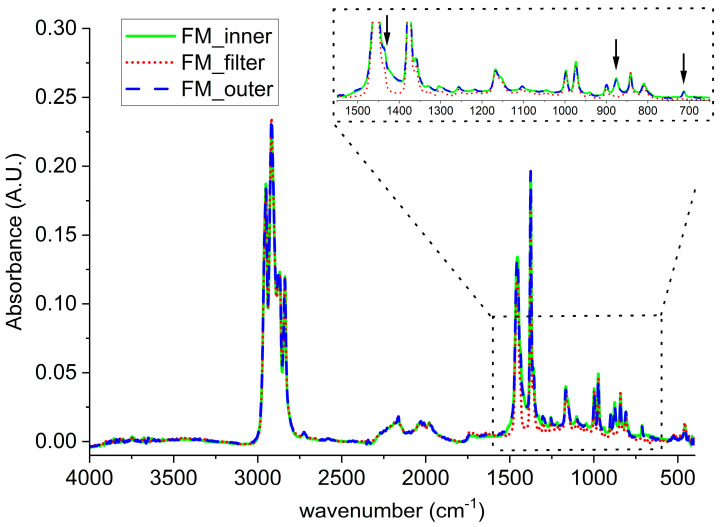
ATR analyses on: (red) FM_filter; (blue) FM_outer; (green) FM_inner part of the disposable mask.

Considering the FMs, the spectrum of the filter (red), the outer (blue), and the inner (green) layers of MCA are reported in Figure 3. All the three are characterized by the typical absorptions of PP as reported in the literature [22].

It worth noting that, for both FM_outer and FM_inner, some absorbance bands differs from the ones of FM_filter (see inlet in Figure 3), specifically, in the area between 1550 cm^−1^ and 1400 cm^−1^ and for two peaks at 876 cm^−1^ and at 712 cm^−1^. Those differences can be attributed to the presence of calcium carbonate, as already revealed in the SEM investigation [23,24].

The ELs present on the market show a great variability in terms of both materials and shape. Notably, in this paper, the attention has been focused on one type (MCA) but, as a minimum survey knowledge, two other types were analyzed to be aware of any problems related to the joint recycling of these parts. The FM of the different masks was analyzed and does not show important differences from that reported previously.

On the other hand, from a structural point of view, the three ELs analyzed were made up of two components: a fabric (coded EL_Type I) and an elastic part (coded EL_Type II). For MCA, the two kind of woven fibers were distinguished by SEM magnification. The same structure can be referred to the EL of the mask coded MCP. Conversely, the components of the mask coded MCD were arranged with a wide single elastic element (EL_Type II) covered with an outer fabric (EL_Type I).

**Figure 4:**
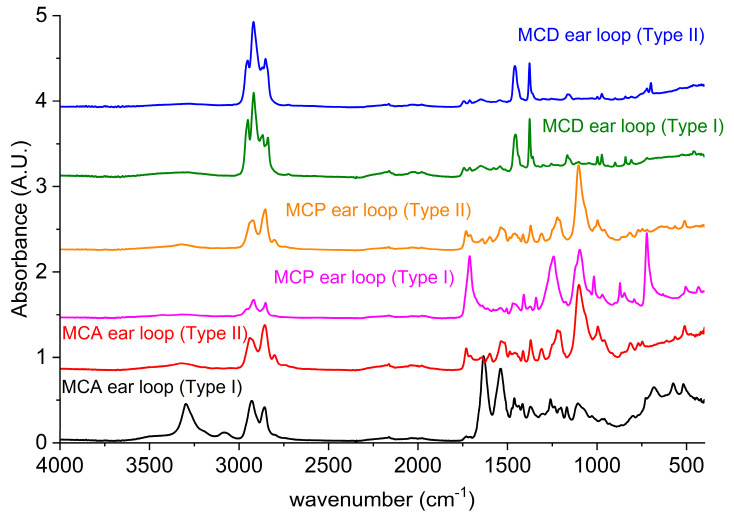
ATR of the disposable face masks from Jointown International from Jinan Shangrun Tongda Composite Material Co., Ltd., Standard GB/T 32610-2016 (MCA); Hubei Hongkang Protective Products Co., Ltd. (MCP); and Oxymore Hangzhou Yuhong Sanitary Products Co., Ltd. (MCD), with the ear loop part divided in EL_Type I and EL_Type II for each mask.

All the above components were investigated with ATR technique, and the spectra are reported in Figure 4. Referring to MCA, the main structure EL_Type I has the IR spectrum typical of polyamide 6 (PA6) [25]. The presence of nitrogen in the EDX elementary analysis then confirms the hypothesis of a PA6.

On the other hand, the fibers named as EL_Type II differed from EL_Type I in the morphology (SEM) and in the chemical analysis (EDX). In addition, the ATR confirms that they are different from EL_Type I and, from the peaks analysis, it can be stated that they are made of elastane [26], a polyurethane-based material well known for its elastic properties. Firstly, a characteristic absorption band is the broad shoulder at about 3300 cm^−1^, due to N-H of the urethane group [27]. At 2940 cm^−1^ and 2856 cm^−1^, the C–H stretching of the aliphatic group on the chain is also detected [28]. Moreover, at 1731 cm^–1^, the band distinctive of C=O stretch is found [29]. Other characteristic peaks are the one referred to C–O stretching at 1101 cm^−1^ [26] and at 1220 cm^−1^, which can be attributed to C–N stretching [28].

Furthermore, in MCP, the EL_Type I (pink curve in Figure 4) was identified as made of Polyethylene terephthalate (PET) [30] for the typical absorption bands at 1717 cm^−1^ attributable to C=O stretching [31], 1245 cm^−1^ related to the aromatic C–C–O asymmetric stretching [30,31], 1102 cm^−1^ correlated to aliphatic O–CH_2_–CH_2_ asymmetric stretching [30,31], and the peaks at 873 cm^−1^ and 724 cm^−1^ due to the presence of C–H on the aromatic rings [30]. On the other hand, the MCP EL_Type II (orange curve) is practically superimposable to the EL_Type II of the MCA (red curve); therefore, it is, again, elastane.

The ear loop of MCD mask, instead, has a different physiognomy: an elastic plane core (coded EL_Type II blue in Figure 4) and a cover of non-woven fabric (EL_Type I green in Figure 4).

The ATR spectra of both the EL_Type I and the EL_Type II of MCD are similar to the non-woven fabric of the filtering part spectra in Figure 3, thus being assigned to PP.

The plastic part of the NW of MCA was analyzed (supporting information Figure S3), and the spectrum was perfectly overlapping the material acknowledged as PP.

#### 3.1.3. Thermal Analyses

**Figure 5:**
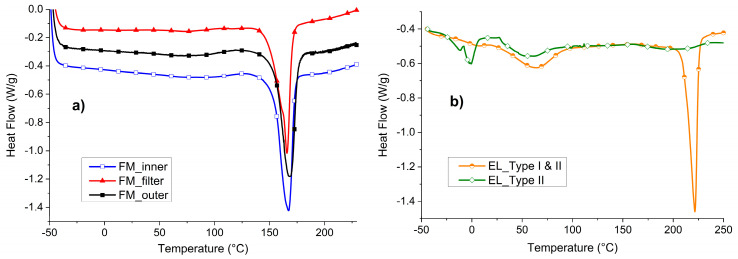
Differential Scanning Calorimetry (DSC) of: (**a**) MCA FM parts and (**b**) EL parts.

In Figure 5, the DSC of the three FM parts of the MCA mask (Figure 5a) and of the two EL types (Figure 5b) are reported.

In Figure 5a, it is possible to appreciate that all the three layers of the FM show one peak of melting in the range between 166 and 168 °C. These temperatures are comparable with the one of PP [32] and in accordance with what was assumed in the ATR analysis. The crystallinity of the three materials is similar: 35% for the external layers and 31% for the filtering part.

In Figure 5b, the DSC of the EL is shown. In particular, the whole ear loop (EL_Type I and II) was compared to the separated EL_Type II fibers. In the case of EL_Type I and II, two main peaks can be distinguished at 64 °C and 222 °C. In accordance with the consideration made with ATR analyses, the one at higher temperature can be attributed to PA6 [33]. On the other hand, the peak at lower temperature in EL_Type I and II has been found to coincide with the peak of EL_Type II and thus can be attributed to the presence of elastane.

### 3.2. Designing the Material Recycling Strategy

**Figure 6:**
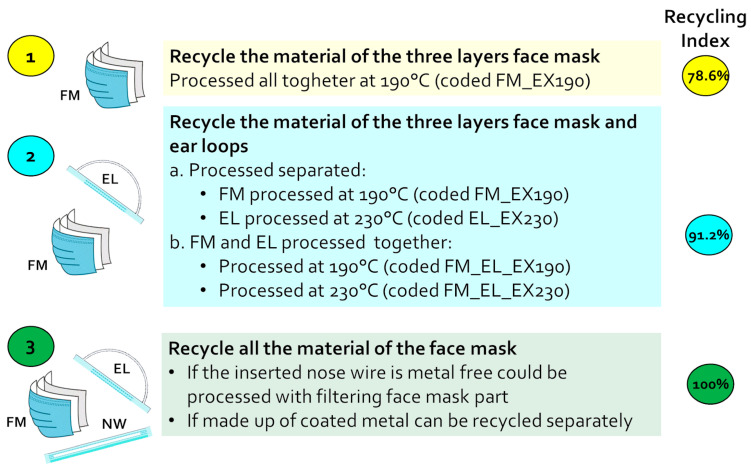
Disposable mask material recycling strategy scheme.

The main aim of this study was to find a way to process the exhausted disposable mask in order to obtain a recycled raw material with properties suitable for an industrial exploitation. With this purpose, the different parts of the mask were processed in different combinations and conditions to evaluate more options. The recycling strategies are schematized in Figure 6, and the detailed mask recycling processes are reported in the Materials and Methods, Section 2.2.

In a first hypothesis (Figure 6, Strategy 1), the material of the FM (78% of the mask weight) was recycled alone (coded FM_EX190). This part is easily separable from the rest, even with an industrial flotation method. Furthermore, even its drying after separation may not be so drastic since the material, PP-based fibers, is not sensitive to humidity during the processing.

In a second strategy (Figure 6, Strategy 2), the EL was also included to increase the recycling rate of the mask to 91%. The route can be carried out with two different approaches (Figure 6, Strategy 2a,b).

In Strategy 2a, connected to the previously presented separation process, the ELs were processed separately from the FM parts (coded as EL_EX230). As the material is mainly a water-sensitive polymer (PA for MCA or PET for MCP), a deeper evaluation would have to be done for the sustainability of the drying process. Indeed, the material requires, first, a deep drying step to eliminate residual moisture due to the separation process or environmental conditions.

In Strategy 2b, the ELs were recycled together with the FMs without any drying process. In this case, the separation process would be extremely simplified by the metal removal of the NW.

Two extrusion temperatures were selected based on the analyses carried out on the materials: 190 °C, in which only the FM (PP) is melted, and the EL acts as filler inside it (coded as FM_EL_EX190); and 230 °C, where the PA6 of the EL will also be in the molten state (coded as FM_EL_EX230).

In Strategy 3, the NW (9% of the mask weight) is taken into account. If the insert is made of only plastic (e.g., PP), it can be recycled together with FM (Strategy 1). On the other hand, if the NW has a metal core that is hardly separable, this fraction could be transferred to the metal recycling circuit. In both cases, 100% of material recycling is achieved.

In this paper, only recycling Strategy 1 and 2 are considered.

### 3.3. Feasibility Evaluation of the Material Recycling Strategy through Thermogravimetric Analysis

**Figure 7:**
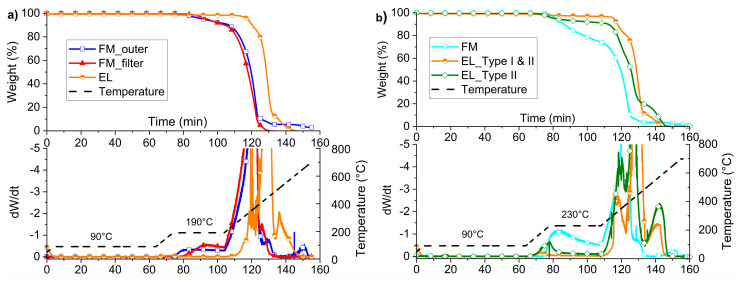
Thermogravimetric Analysis (TGA) of (**a**) FM_outer, FM_filter, and EL with processing simulation at 190 °C (weight % up/derivate down); (**b**) TGA of FM, EL_ Type I and II, and EL_Type II with processing simulation at 230 °C (weight % up/derivate down).

The thermal stability in oxidative atmosphere was analyzed for both the FM layers and the EL, in order to evaluate the effect of the recycling conditions on the materials and verify the technical feasibility. In particular, the drying temperature of 90 °C and the selected processing temperatures of 190 °C (Figure 7a) or 230 °C (Figure 7b) were used for isothermal steps in the TGA analysis, in accordance with Strategy 1 and 2 in Figure 6.

In Figure 7a,b, the thermograms referring to the processing simulation at 190 °C and 230 °C, respectively, are presented.

In Figure 7a, the thermograms of the outer layer, the filter layer, and the EL are presented, along with the speed of variation of the weight and the temperature ramp. As can be seen, all the materials withstand the maintenance at 90 °C for 1 h, but both the mask layers show a weight reduction of about 9 wt.% during the 30 min isothermal at 190 °C. The degradation rate of FM_outer is higher at this temperature than the FM_filter, as the derivative curve rises after about 2 min of isotherm in FM_outer, while, in the MCA_filter, after about 6 min. On the other hand, the ear loop starts to degrade in the heating ramp just over 270 °C.

Referring to the residue at 600 °C, the FM_outer showed a residual weight of about 4 wt.%, while no residue was observed neither for the FM_filter nor for the EL. The difference in the residue of filter and outer mask layers is attributable to the presence of calcium carbonate, as discussed both in ATR analysis and morphological investigation.

The general behavior of the FM_outer and FM_inner are comparable; thus, the inner part is not reported.

Thanks to the data collected by TGA, it can be stated that: (1) it is possible to dry at a temperature of 90 °C for 1 h without having experimental evidence of weight loss of all the materials of the masks; (2) the processing at 190 °C is tricky for PP of the masks, which begins to lose weight already after 2 min. The maintenance times in temperature must be limited as much as possible. The recirculation time in the micro extruder was thus chosen in 2 min not to have an excessive degradation, but to be comparable to the residence times of the materials in industrial extruders. On the other hand, the material of the EL presents no problems for the process at this temperature.

The thermoxidative stability at the highest isothermal settings (230 °C) is generally poorer than the previous one (Figure 7b). The three FM layers, examined together this time, show a weight loss of about 24 wt.% at the end of the isothermal step of 30 min. As expected from the previous test, the degradation already begins before the isothermal phase but is not so dramatic (2%). Obviously, 30 min of processing at this temperature is unlikely, but the analysis was done to analyze the kinetics of this degradation. It turns out to be quite constant in the first minutes with a weight loss rate of about 1 wt.% every minute.

Moreover, during the processing simulation at 230 °C, EL_Type I and II is affected by a weight reduction of about 1 wt.%, which achieves almost 5 wt.%, considering just EL_Type II.

No noticeable variations of the residue at 600 °C have to be mentioned compared to the results with the isotherm at 190 °C.

It is possible to state from this second TGA analysis campaign that the PP of FM and EL_Type II of the mask under consideration show an onset degradation temperature before 230 °C, while the EL_Type I part is thermally the most resistant. For this reason, recycling at this temperature is risky for FM, but not impossible, since the weight loss is not so extensive. The separation of the two components of the EL is hardly possible only manually, but impracticable in an industrial vision, as they are woven together. Some research studies have been carried out for the chemical separation of elastane from PA6, but they appear hardly industrially applicable [34,35]. For this reason, the joint recycling is imagined, hoping that the low weight percentage of the EL_Type II may not be so dramatic for the whole properties.

### 3.4. Characterization of the Recycled Materials

Defining the recycling procedures and their technical feasibility, the four materials obtained (FM_EX190, EL_EX230, FM_EL_EX190, and FM_EL_EX230) were characterized and the results are reported in this section.

#### 3.4.1. Morphological Analyses

**Figure 8:**
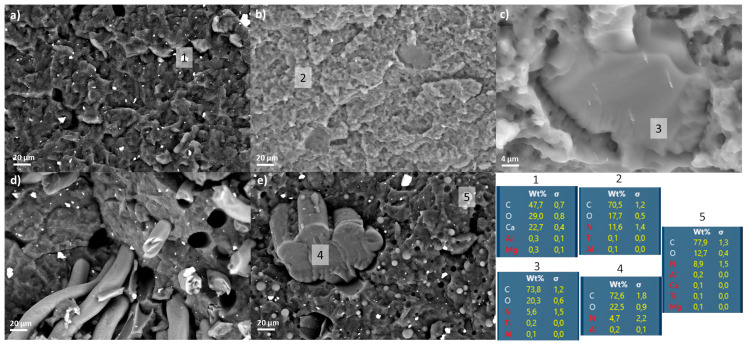
SEM images of: (**a**) FM_EX190, (**b**,**c**) EL_EX230, (**d**) FM_EL_EX190, and (**e**) FM_EL_EX230.

The material resulting from the recycling of the FM (Figure 8a) appears as a continuous matrix of PP in which calcium carbonate particles are uniformly dispersed as predictable from the preliminary analyses on the pristine masks.

Similarly, the morphology of the EL extruded separately at 230 °C (Figure 8b) is a continuous matrix with the presence of isolated structures not so obvious at first glance. The high magnification picture (Figure 8c) highlights the smoother surface of such structures with respect to the corrugated outline of the matrix. In addition, white dots are visible inside this central smooth area and the elementary analysis revealed the presence of titanium element as had been highlighted by the analyses on the fibers. Furthermore, the analysis of the nitrogen element in the matrix (11.6 ± 1.4 wt.%) and central area (5.6 ± 1.5 wt.%) confirms the ratio between the EL_Types I and II. Finally, the dimensions of this central area are in the order of 40 microns, similar to the dimensions of a single EL_Type II fiber (Figure 2).

Focusing on the fracture surface, it appears to be on the same level with respect to the matrix (Figure 8c). This fact is generally a symptom that there is a good adhesion between the two materials; therefore, the mechanical stresses can be transmitted from one phase to the other.

In order to deepen the evaluation of interaction between FM and EL processed together, the materials obtained at 190 and 230 °C were analyzed on a morphological point of view and compared to the materials processed separately (Figure 8d,e).

In the FM_EL_EX190 sample, the fibers of the EL are still present both in strands and single fibers (Figure 8d). Moreover, as can be seen from the gaps at the interface and the fiber pull-out (Figure 8d), the adhesion of the matrix on the EL is very poor. It worth noting that the particles of calcium carbonate already seen in the FM_inner and FM_outer are also distinguishable in the matrix material (Figure 8d).

On the other hand, the compound extruded at 230 °C (Figure 8e) shows a different morphology: in addition to the calcium carbonate particles, spherical particles can be seen in the matrix, and the EL strands are few. The EDX analysis highlights that the concentration of nitrogen in the spheres (8.9 ± 1.5 wt.%) is comparable with the one of EL_Type I fibers in EL_EX230. For this reason, and in accordance with the DSC results, it can be concluded that these spherical formations are due to the melting of the EL_Type I fibers that, because of the poor compatibility with the matrix, arranges in spheres when cooled. Consequently, no more intact EL_Type I fibers can be seen in the material, but only few solid joint fibers of the original EL_Type II fibers, partially melted together (Figure 8e). In addition, in this case, the elemental analysis on this structure reveals the presence of 4.7 ± 2.2 wt.% of nitrogen, which is comparable to that found in the separated structures of EL_EX230 sample.

#### 3.4.2. Rheological Analyses

**Figure 9:**
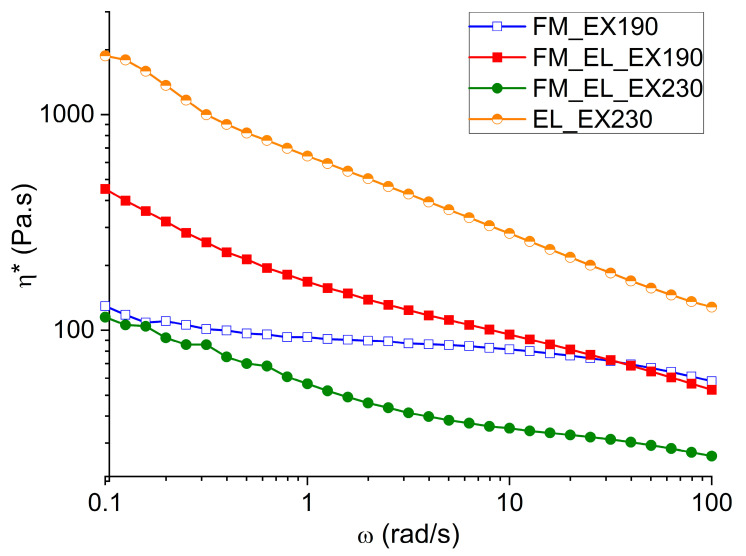
Complex viscosities at 190 °C of FM_EX190, FM_EL_EX190, EL_EX230, and FM_EL_EX230.

The materials extruded and compression molded were characterized in terms of complex viscosity (η*) in order to compare the mask recycled materials with commercial ones and evaluate the possible production processes. In Figure 9, the η* at frequency sweep between 0.1 and 100 rad/s at 190 °C or 230 °C can be seen.

The sample FM_EX190 exhibits a non-Newtonian behavior typical of polymeric materials with a Newtonian plateau at around 150 Pa∙s and, finally, a zone of shear thinning at low frequencies due to the presence of the well dispersed calcium carbonate filler. The value of η* at 0.1 rad/s is 174 Pa∙s. Compared to other PPs, the η* is about an order of magnitude lower [36]. These differences can be attributable to a lower molecular weight in the case of the material obtained from the masks. This general behavior of the material was also confirmed by melt flow rate (MFR) measurement, which gave as a result 55 ± 4 g/10 min at 190 °C and 2.16 kg of load.

In addition, the EL_EX230 shows the shear thinning behavior in the η* at low frequencies typical of the presence of second phase or a filler (Figure 9). As it has been observed from the morphological analysis, the microstructure of the EL_EX230 presents the EL_Type II part inside a matrix made of EL_Type I. These un-melted parts behave like a filler and cause the shear thinning trend.

At the same time, the general trend of the η* reflects the one proper of PA6, both referring to the Newtonian plateau viscosity value (in the order of 10^3^ Pa∙s), both in the decreasing of the η* value with shear rate [37,38,39,40].

Considering the combined recycling of both parts at 190 °C, the addition of the EL in FM matrix can be clearly seen in a stronger shear-thinning behavior. Indeed, the processing and testing temperature of 190 °C is too low to obtain the melting of the EL; thus, all the chopped fibers last in the PP-matrix, like a filler. The η* at 0.1 rad/s is 174 Pa∙s for the pristine matrix and 453 Pa∙s with the presence of the chopped EL. As far as the highest processing temperature of the joined recycling was concerned (FM_EL_EX230), the EL_Type II fibers are preserved, while the EL_Type I are transformed into spheres; thus, a change in the viscosity trend is foreseeable. The η* is generally shifted to lower values as the analysis temperature is now at 230 °C, and the shear thinning part is less pronounced due to the presence of the spheres and fewer structures made of fibers. The η* at 0.1 rad/s is 115 Pa∙s, the lowest among the analyzed samples.

#### 3.4.3. Mechanical Properties

**Figure 10:**
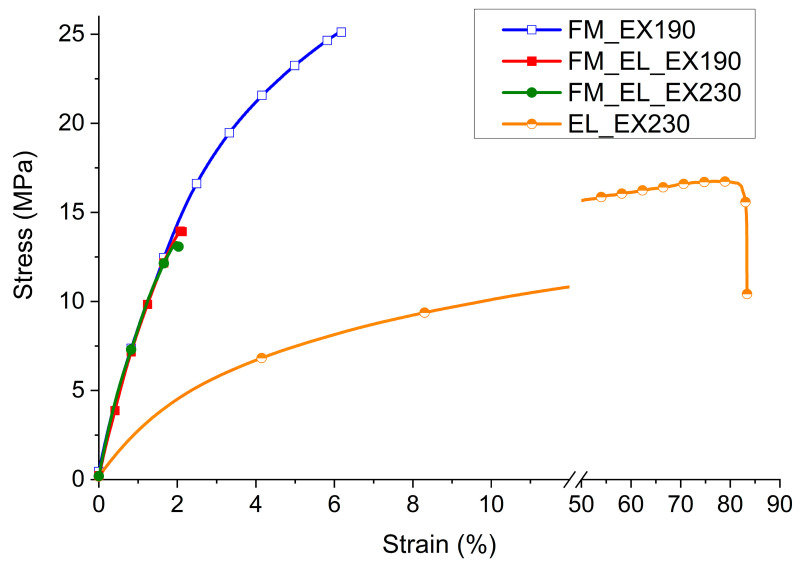
Stress-strain curves from tensile tests of FM_EX190, FM_EL_EX190, FM_EL_EX230, and EL_EX230.

The recycled materials were analyzed on a mechanical point of view in the form of a film with a thickness of about 100 microns. The stress-strain curves in Figure 10 were selected as the sample more in accordance with the average values within the single material and the average values of the above parameters are listed in Table 1.

Comparing the FM_EX190 with typical PP copolymer values [32], it emerges that the recycled material has very similar behavior in terms of maximum stress (24 ± 2 MPa vs. 20–30 MPa) and Young’s Modulus (914 ± 12 MPa vs. 800–1300 MPa), while it strongly differ for the elongation at break (5 ± 1% vs. >50%). This low elongation at break behavior is similar to what is found for commercial recycled materials (e.g., 10% for EKOBOARD Recycled by Ekon B.V., Born, The Netherlands).

The stress-strain curve of EL_EX230 conditioned at 50% of R.H. has a modulus of 250 MPa, a maximum stress of 17 ± 1 MPa with an elongation at break over 80%. A modulus between 750–1500 MPa, maximum stress of 30–60 MPa, and an elongation at break >50% and until 200% are reported for a PA6 in a polymer handbook at the same environmental conditions [32].

The material is, therefore, much weaker than a reference first-use PA6 sample, but it has still a good deformability and flexibility. On the other hand, considering a post-consumer recycled PA6 (e.g., EnViramid^®^ N2300STHL BK from Ravago Manufacturing Americas (Orlando, FL, USA): strength at break of 25 MPa and a strain at break of 55%), the values are much closer.

As can be further seen in the same plot in Figure 10, the FM_EL_EX190 and FM_EL_EX230 samples have properties extremely different from the material processed separately, FM_EX190 or EL_EX230. For both the extrusion temperatures (190 °C and 230 °C), a slight increase in the Young’s Modulus, along with a significant reduction in the max strength (−41%) and strain at break (−71%), were observed.

These properties can be connected to the morphological evidences revealed by SEM analyses. In particular, the similarity between the characteristics of the compound extruded at the two different temperatures testifies that, from a mechanical point of view, it is more relevant the poor compatibility between PP of the protective layers and the PA of the ear loop than the different morphology obtained.

#### 3.4.4. Thermo-Mechanical Properties

**Figure 11:**
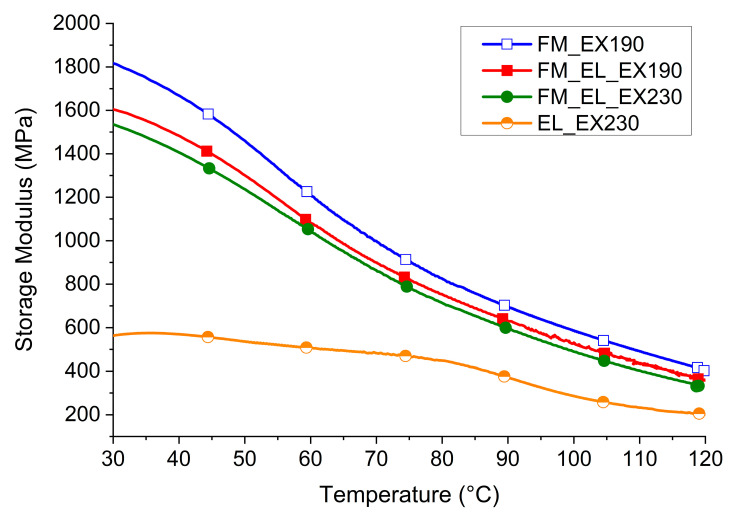
DMTA of FM_EX190, FM_EL_EX190, FM_EL_EX230, and EL_EX230.

The dynamo mechanical behaviors of the materials in temperature ramp were examined and are reported in Figure 11. The test was carried out to verify the applicability of the materials at temperatures higher than room conditions. The general trend of the materials based on the PP matrix is in accordance with the one expected as described in other research studies [41,42]. A storage modulus of 800 MPa is still measured over 70 °C for the three samples (see Table 1). On the other hand, the EL_EX230 sample has a different behavior because its highly sensibility to moisture. The tested sample was conditioned at 50% of R.H., which moves away from the sample during the test. For this reason, the modulus at low temperatures is not very high (<600 MPa) but is better maintained in temperature as the plasticizing effect of the water in the matrix decreases with temperature.

### 3.5. Summary of the Recycling Strategies

The main results of the analyses and the developments and applications for every material obtained following the different suggested strategies are summarized below.

Recycle the material of the three layers face mask (Strategy 1):

- The PP recycled from the masks has a very low complex viscosity.
- The tensile tests found that the elongation at break is extremely lower than “standard” PP, while the tensile modulus and strength are comparable.
- The application in temperature of the material is comparable with what is expected for a PP.

The recycled material can be exploited in a mixture with a first use PP with higher viscosity for the direct use in application as injection molding or extrusion. A second possibility is the addition of a filler from wastes (e.g., agro-industrial source), probably even in a considerable amount, given the low complex viscosity of the recycled mask material.

Possible applications are material not subjected to shocks or significant deformations but able to withstand loads (e.g., furniture or other domestic appliances).

Recycle the material of the ear loops (Strategy 2a):

- The PA recycled from the ear loops has a complex viscosity comparable to a PA6.
- The tensile performances reveal properties less than 50% of a common PA6.

The recycled material can be exploited directly in injection molding for applications where high mechanical characteristics are not required. Furthermore, the addition of specific filler could give notable property improvements.

The drawback regarding recycling the EL part of the masks lies with the heterogeneity due to the presence of two types of fibers made of several types of materials commercialized.

Recycle the material of the three layers face mask and ear loops together (Strategy 2b):

- This solution gives the worst mechanical results among those analyzed.
- The application in temperature of the material is still feasible.
- The variation in the processing temperature (190–230 °C) does not give significant changes in the mechanical and thermo-mechanical properties.

This solution is the simpler and probably the cheapest, but it needs extensive studies to improve the properties. Enhancement can be expected with the use of compatibilizers and other fillers.

## 4. Conclusions and Outlook

A detailed characterization of the mask material was carried out. From the thermal, morphological, and chemical analyses, different recycling strategies were proposed, validated, and two of them developed.

The main conclusion from the research presented answers the question reported in the title. The materials of the disposable masks can be mechanically recycled, and the four materials obtained from recycling Strategies 1 and 2 have the potential for industrial exploitation. Taking into account the considerable heterogeneity of the commercialized mask materials, different strategies could be introduced, and new questions need other investigations. The requirement to improve the performances of the recycled materials has emerged; thus, investigation on blends with first-use polymers or the addition of fillers, compatibilizers, and additives are foreseeable. Nevertheless, this article could be a milestone for future development in this topic due to the rise of scientific interest in personal protective equipment recycling.

## Figures and Tables

**Table 1:** Average values of Young’s Modulus (E), maximum stress (σ_M_), and strain at break (ε) from tensile tests and average temperature corresponding to 800 MPa of storage modulus from dynamic-mechanical thermal experiments (DMTA) of FM_EX190, FM_EL_EX190, FM_EL_EX230, and EL_EX230.

Material	Average E (MPa) (Standard Deviation)	Average σ_M_ (MPa) (Standard Deviation)	Average ε (%) (Standard Deviation)	Average Temperature at Storage Modulus of 800 MPa (°C) (Standard Deviation)
MCA_EX190	825 (50)	22 (1)	7 (1)	80 (1)
MCA_Ela_EX190	921 (17)	13 (3)	2 (0)	77 (1)
MCA_Ela_EX230	1049 (95)	13(2)	2 (0)	72 (2)
Ela_DRY_EX230	253 (8)	17 (1)	84 (9)	-

## Supplementary Materials

The following are available online at https://www.mdpi.com/2073-4360/12/11/2726/s1, Figure S1: EDX of EL_Type I and EL_Type II, Figure S2: EDX of NW, Figure S3: ATR of the surface of the NW.
