# Supplementary material for: Is it Possible to Mechanical Recycle the Materials of the Disposable Filtering Masks?
Source: Polymers (Basel). 2020 Nov 17;12(11):2726. doi: 10.3390/polym12112726 (PMC7698554; doi:10.3390/polym12112726)
Supplement: Supplementary file 1 [file polymers-12-02726-s001.pdf]

polymers

MDPI

Article

Is it Possible to Mechanical Recycle the Materials of the Disposable Filtering Masks?

Daniele Battegazzore *, Fulvia Cravero and Alberto Frache

Dipartimento di Scienza Applicata e Tecnologia, Politecnico di Torino, Alessandria site,
Viale Teresa Michel 5, 15121 Alessandria, Italy; fulvia.cravero@polito.it (F.C.); alberto.frache@polito.it (A.F.)
* Correspondence: daniele.battegazzore@polito.it, Tel/Fax: +390131229343/+390131229399

Supporting Information

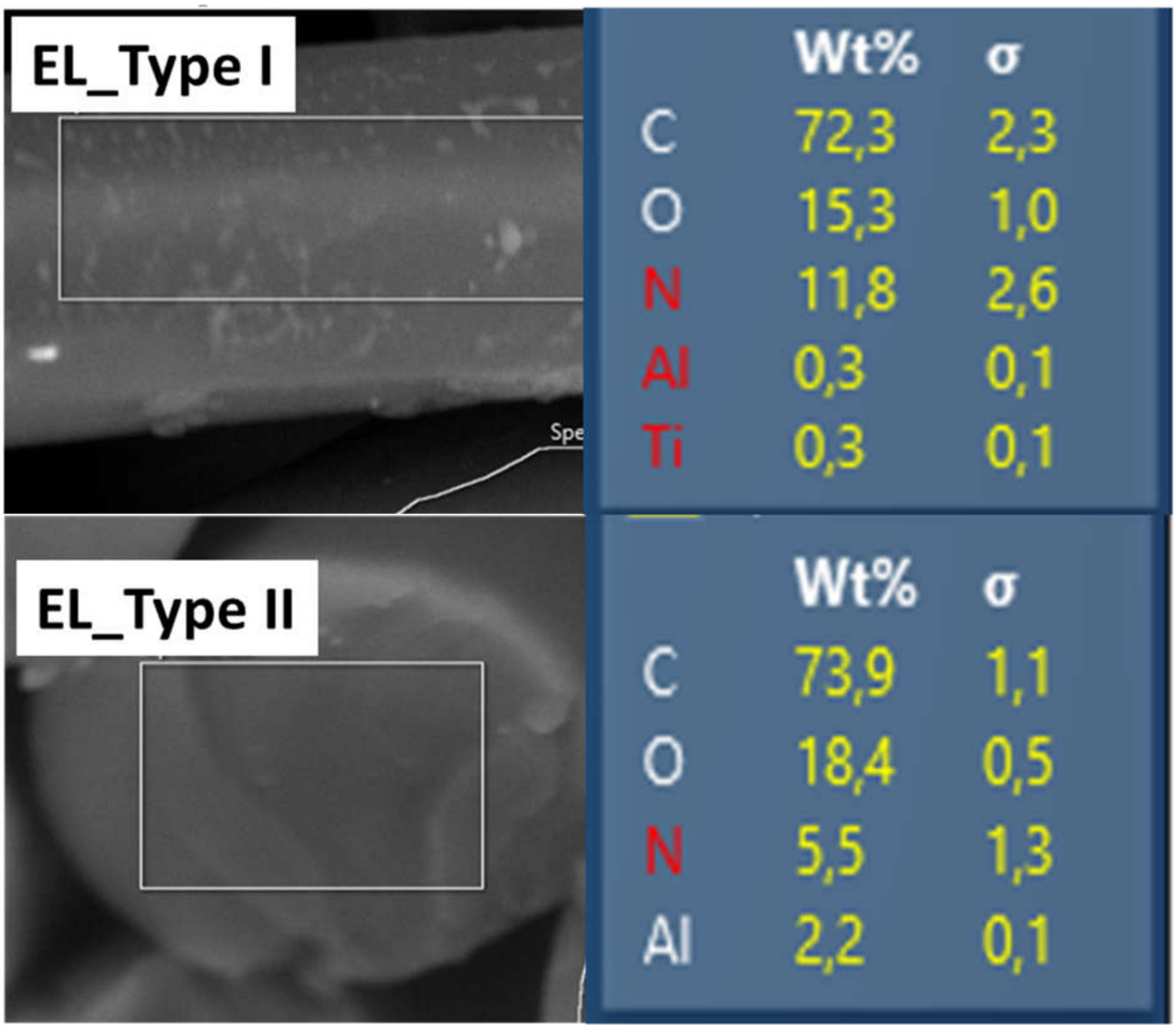

Figure S1. EDX of EL_Type I and EL_Type II.

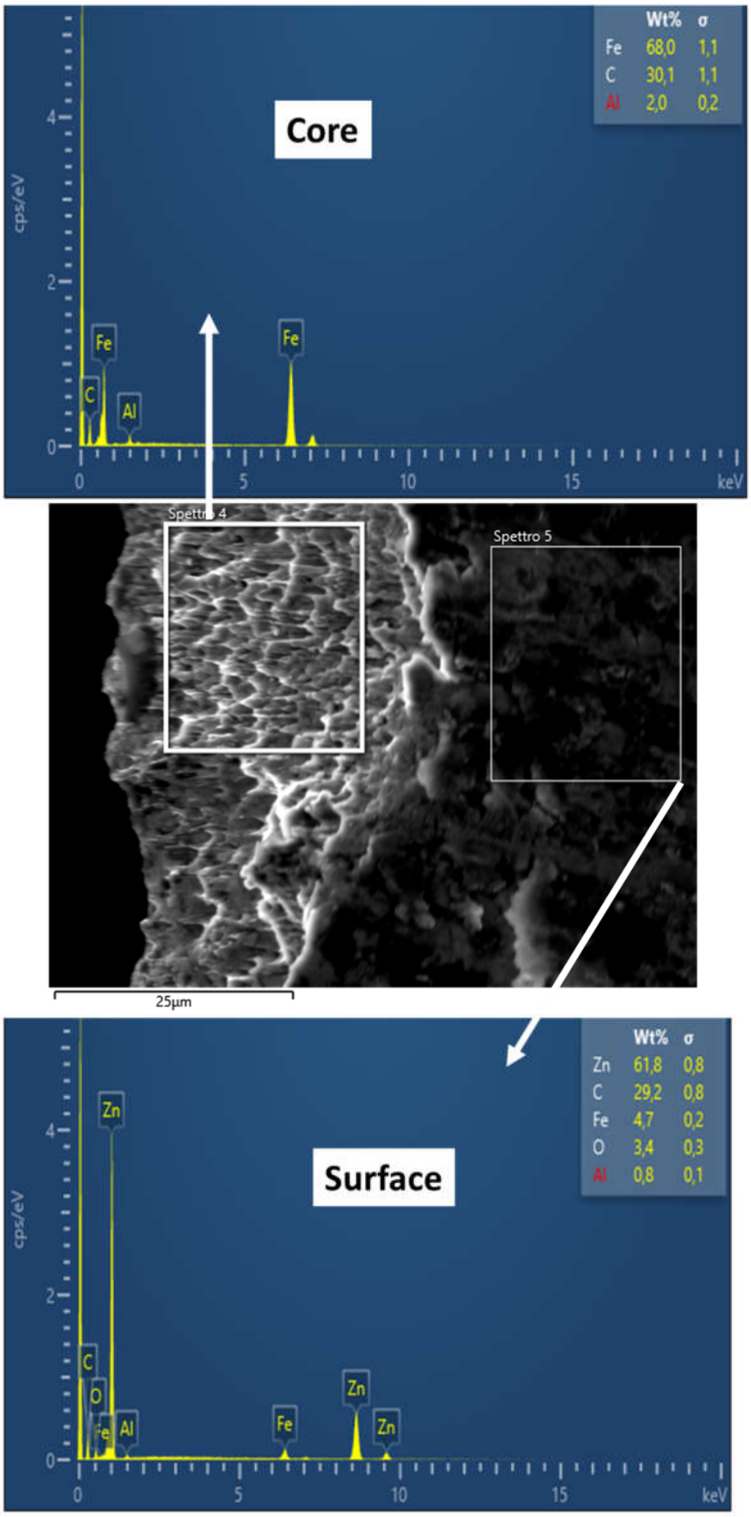

Figure S2. EDX of NW.

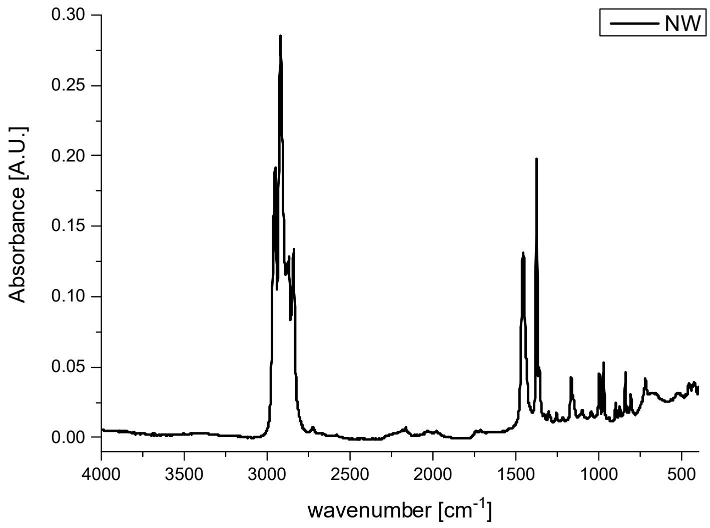

Figure S3. ATR of the surface of the NW.
